# Assessment of Headache in Asthma Patients

**DOI:** 10.12669/pjms.331.11720

**Published:** 2017

**Authors:** Adil Can Gungen, Belma Gungen

**Affiliations:** 1 Adil Can Gungen, MD. Department of Pulmonology, Sakarya University, Education and Research Hospital, Sakarya, Turkey; 2 Belma Gungen, MD. Department of Neurology, Sakarya University, Education and Research Hospital, Sakarya, Turkey

**Keywords:** Asthma, Headache, Migraine

## Abstract

**Background and Objective:**

Headache is a common health problem, which may present with neurological diseases and other chronic diseases, and has an adverse effect on the emotional status. We think that headache is a common disease in asthmatic patients. This study aims to evaluate the presence of headache and risk factors in patients with asthma.

**Methods:**

Ninety-three patients with asthma and 58 healthy control subjects were included in the study. The presence of headache was evaluated according to the revised criteria of the International Classification of Headache Disorders, 2nd edition (ICDH-II). Asthma control test (ACT) was performed to determine asthma control status. The Beck Depression Inventory (BDI) and Beck Anxiety Inventory (BAI) were performed in all participants. Demographic features, used medications, and presence of headaches were recorded.

**Results:**

Fifty-eight patients with asthma (62.4%) had headaches, whereas only 19 control subjects (32.8%) had headaches. Thirty-two patients (34.4%) had tension-type headache, 19 patients (20.3%) had migraine-type headache, and 7 patients (7.5%) had other types of headaches. The frequency of headaches was significantly higher in patients with asthma, compared to healthy control subjects (p=0.001). There was a significant correlation between migraine-type headache and inhaled steroid use, and presence of allergies.

**Conclusion:**

Migraine-type and tension-type headaches are more common in patients with asthma, compared to the overall population. The frequency of migraine-type headache is higher in patients with asthma who have allergies and low respiratory function test scores.

## INTRODUCTION

Asthma is characterized by recurrent wheezing, difficulty in breathing, airway hyper responsiveness that causes coughing attacks. The diagnosis is based on anamnesis, spirometric examination, physiological and pathological findings.^1^ Asthma affects approximately 300 million individuals worldwide, and results in 250,000 asthma-related deaths. The prevalence of asthma ranges between 1% and 18% among countries.^2^-^4^ Several genetic and environmental factors play a role in development of asthma.^5^,^6^

Asthma can be accompanied by different diseases (including but not limited to allergic rhinitis, obesity, and gastroesophageal reflux) which have a negative effect on asthma and affected individual. Co-existence of asthma and psychiatric diseases (particularly panic attack; also including anxiety disorders and depression) has been demonstrated in different studies.^7^-^9^

Headache is one of the most common complaints, which results in loss of workforce and poor life quality. International Headache Society (IHS) diagnostic criteria are used to classify headaches.^10^ Tension-type and migraine-type headaches are the most common types. Headache can accompany neurological diseases, as well as systemic diseases, including hypertension, cardiac pathologies, fibromyalgia, irritable bowel syndrome, and systemic lupus erythematosus.^11^-^13^

The relationship between asthma and headache has been demonstrated in only a small number of studies.^14^-^18^ On the other hand, in some of these studies, diagnosis of asthma was based on questioning and presence of symptoms. In addition, the factors affecting headache in patients with asthma were not clear. To our knowledge, this is the first study to explore the presence of headaches, causes underlying headaches, and types of headaches in patients who are followed up with asthma diagnosis, and who do not have comorbid chronic diseases.

## METHODS

A total of 93 patients who were diagnosed with asthma according to the Global Initiative for Asthma (GINA) criteria at Sakarya University Hospital, Turkey between March 2014 and April 2015 were included in the study. The control group consisted of 58 healthy control subjects, who were similar with respect to age, sex, and educational status. The study received ethical approval from institutional Ethics Committee. An informed consent was obtained from each participant. The study was conducted in accordance with the principles of the Declaration of Helsinki.

All patients underwent physical and neurological examination. Exclusion criteria were as follows: the presence of comorbidities, using nitrate and MAO inhibitors, alcoholism, using oral or parenteral steroids for asthma, using sedatives, hypnotics and antidepressants, having pathological opthalmic findings, secondary headache, and illiteracy. The presence of headache was evaluated, and classified according to the revised criteria of the International Classification of Headache Disorders, 2nd edition (ICDH-II).^10^ The types of headaches were classified as follows: Migraine-type, tension-type, autonomic, and others. Asthma control test (ACT) was performed by the same pulmonologist to control the disease status. The results of respiratory function tests were evaluated. Patients were questioned for history of allergies. The Beck Depression Inventory (BDI) and Beck Anxiety Inventory (BAI) were used to evaluate anxiety and depression levels. Correlations between inhaled steroid use, β2 mimetic use, IgE, sex, ACT score, SFT values, depression, anxiety, and presence of headache/type of headache were investigated.

For the evaluation of emotional status, BAI and BDI were used.^19^,^20^ These scales are widely used, 21-item standardized, self-administered questionnaires which measure various symptoms of anxiety- depression and describe the somatic and cognitive-affective symptoms on a four-point scale that ranges from 0 to 3. A higher score indicates more severe anxiety-depression and in this study, the cut-off point was ≥17 points for BAI and was ≥10 points for BDI.

Asthma Control Test (ACT), the patients were administered a 5-item questionnaire assessing their asthma symptoms, use of rescue medications, and the impact of asthma on daily life. Scores range from 5–25 (higher is better).^21^ The validity and reliability of this questionnaire has been previously shown in adult Turkish patients with asthma.^22^

### Statistical Analysis

Statistical analysis was performed on SPSS v21.0 software (SPSS Inc., Chicago, IL, USA). Normally distributed continuous variables were analyzed using samples t-test, while non-parametric variables between the two groups were analyzed using the Mann–Whitney U test. Categorical variables were compared using the chi-square test. A general linear model was used to correct for emotional status and sex. Multivariate regression analysis was used to evaluate the effective factors. A p value of <0.05 was considered statistically significant.

## RESULTS

A total of 93 patients with asthma and 58 healthy control subjects were included in the study. Demographic features, clinical characteristics, and laboratory and respiratory function test results of all participants are shown in Table-I. There was no significant difference in the age, sex, and hemoglobin levels between the two groups (Table-I).

**Table-I:** Demographic and clinical characteristics, laboratory and respiratory test function results.

	Asthma (n=93)	Controls (n=58)	P value
Sex (F/M)	(62 /31)	(41/17)	0.295
	*Mean±S.D.*	*Mean±S.D.*	
Age	48.48 ± 11.6	46.3 ±9.1	0.063
Hemoglobin(gr/dl)	13.4 ±1.12	13.4±1.1	0.751
IGE (IU/ml)	182.3±263	-	
Age at onset	41.2±13.2	-	
Disease duration	7.15±7.42	-	
Number of attacks (year)	2.1±1.4	-	
ACT	17.5±5.2	-	
FVC (%)	85.01±17.7	-	
FEV1 (%)	77.2±19.1	-	
FEV1/FVC	77±7.9	-	
PEF (%)	77.4±22.5	-	

ACT, Asthma Control Test™; FVC, forced vital capacity; FEV_1_, forced expiratory volume in 1 second; PEF, peak expiratory flow; IgE, immunoglobulin E.

Fifty-eight patients with asthma (62.4%) had headaches, whereas only 19 control subjects (32.8%) had headaches (p=0.001). The frequencies of migraine-type and tension-type headache were significantly higher in patients with asthma, compared to control subjects (p=0.04 and p=0.03, respectively). In addition, the frequencies of anxiety and depression were also significantly higher in patients with asthma (p=0.002 and p=0.008, respectively) (Table-II).

**Table-II:** Comparison of headache, headache types, and emotional state between patients with asthma and control subjects.

	Asthma patients (n=93)	Control subjects (n=58)	P value

	N (%)	N (%)	
Presence of headache	58(62.4)	19(32.8)	0.001
Migraine-type headache	19(20.3)	5(8.6)	0.04
Tension-type headache	32(34.4)	11 (19)	0.03
Other types of headache	7(7.5)	3(5.2)	0.41
Depression	36(38.7)	11(19)	0.002
Anxiety	36(38.7)	9(15.5)	0.008
	*Mean±S.D.*	*Mean±S.D.*	
BAI score	13.3±7.9	8.3± 5.3	0.002
BDI score	7.6±5.6	5.7 ±3.3	0.004

BAI: Beck Anxiety Inventory, BDI: Beck Depression Inventory.

In the asthma group, a significant relationship was not found between the presence of headache and inhaled steroid use, β_2_ agonist use, age, immunoglobulin E, forced vital capacity (FVC), forced expiratory volume in 1 second (FEV_1_), FEV_1_/FVC and peak expiratory flow values (Table-III).

**Table-III:** Correlation between headache and age, sex, ACT, inhaled steroid use, β2 agonist use, IgE, FVC, FEV1, FEV_1_/FVC (%) and PEF in patients with asthma.

N=93	Headache present	Headache absent	P value
Sex (F/M)	41 / 17	21 / 14	0.054
	*Mean±S.D.*	*Mean±S.D.*	
Age	48.7±11.4	48.02±12.2	0.560
Disease duration	7.2±8	6.9±6.3	0.324
Age at onset (years)	41.3±13.11	41±13.5	0.649
ACT	17.2±5.3	18.05±5.1	0.817
FVC (%)	82.6±16.3	88.9±19.4	0.161
FEV1 (%)	76.4±18.6	78.8±20.4	0.608
FEV1/FVC (%)	76.8±7.4	77.2±8.7	0.579
PEF (%)	76.3±23.1	79.1±21.7	0.703
IGE	181.8±284	183±248.6	0.873
Beck depression score	13.3±7.6	5.7±4.4	0.036
Beck anxiety score	8.7±6.2	11.4±8.5	0.358
	*n (%)*	*n (%)*	
Inhale steroid use	39 (67.2)	28 (80)	0.138
β_2_ agonist use	53 (91.3)	32 (91.4)	0.652
			

ACT, Asthma Control Test™; FVC, forced vital capacity; FEV_1_, forced expiratory volume in 1 second; PEF, peak expiratory flow; IgE, immunoglobulin E.

In the asthma group, a significant relationship was not found between the presence of tension-type headache and inhaled steroid use, β_2_ agonist use, age, immunoglobulin E, forced vital capacity (FVC), forced expiratory volume in one second (FEV_1_), FEV_1_/FVC and peak expiratory flow values. In the asthma group, a significant correlation was found between the presence of tension-type headache and gender, Beck depresyon score (respectively, p:0.009, p:0.005).

In the asthma group, a significant correlation was found between the presence of migraine-type headache and age, inhaled steroid use, presence of allergies, forced vital capacity (FVC), forced expiratory volume in one second (FEV_1_) and FEV_1_/FVC (p=0.03; p=0.037, p=0.001; p=0.011; p=0.015; p=0.037) (Table-IV).

**Table-IV:** Correlation between migraine and age, sex, ACT, inhaled steroid use, β2 agonist use, presence of allergies, IgE, FVC, FEV1, FEV_1_/FVC (%) and PEF in patients with asthma.

N=93	Migraine present	Migraine absent	P value
Sex (F/M)	11/8	51/23	0.259
	*Mean ± S.D.*	*Mean ± S.D.*	
Age	41.1±11.6	50.3±10.9	0.03
Disease duration	8.8±9.17	7.2±6.8	0.359
Frequency of attacks	2.4±2.2	2.1±1.24	0.920
ACT	16.7 ±5.1	17.7±5.3	0.44
FVC (%)	76.9±17.2	87.08±17.3	0.011
FEV1 (%)	67.2±18.6	79.8±18.5	0.015
FEV1/FVC (%)	73.7 ±7.6	77.8±7.8	0.037
PEF (%)	73.3±22.8	78.4±7.8	0.511
IGE	236±380.5	168.5±225.2	0.289
	*n (%)*	*n (%)*	
Inhale steroid use	10 (52.6)	57 (77.02)	0.037
β_2_ agonist use	16 (84.2)	69 (93.2)	0.205
Presence of allergies	14 (73.6)	21 (28.3)	0.001

ACT, Asthma Control Test™; FVC, forced vital capacity; FEV_1_, forced expiratory volume in one second; PEF, peak expiratory flow; IgE, immunoglobulin E.

The multivariate analysis showed that advanced age, inhaled steroid use, presence of allergies, forced vital capacity (FVC), forced expiratory volume in 1 second (FEV_1_) and FEV_1_/FVC were associated with Migraine. These factors affect the presence of migraine by 30 percent. According to Multivariete analysis; age, presence of allergy and FEV1/FVC affect the migraine significantly in a high percentage (Table-V).

**Table-V:** Multivariate logistic regression model for Migraine.

	β	S.E. of β	p values	OR (95% C.I. for OR)
Age	0.106	0.032	0.001	1.112 (1.044-1.185)
Presence of allergies	1.877	0.701	0.007	6.533 (1.653-25.814)
FVC (%)	0.104	0.062	0.093	1.11 (0.983-1.254)
FEV1 (%)	-0.068	0.061	0.265	0.934 (0.828-1.053)
FEV1/FVC (%)	0.131	0.069	0.05	1.140 (0.996-1.304)
Inhale steroid use	-0.740	0.720	0.304	0.477(0.116-1.958)

FVC, forced vital capacity; FEV_1_, forced expiratory volume in 1 second.

## DISCUSSION

In the present study, we evaluated the presence of headache in patients with asthma according to the IHS diagnostic criteria and classified with respect to types of headache. We also evaluated the correlation between the presence of headache and asthma control status, history of allergies, anxiety, depression, use of inhaled steroids, and respiratory functions. Our findings suggest that migraine and tension-type headache are common in asthma patients. In addition, headaches were predominantly seen in female patients. We also observed a significant correlation between migraine-type headache, allergies, and low respiratory function test results. In addition, the frequency of migraine-type headache was lower in patients receiving inhaled steroids.

In the pathogenesis of co-existence of asthma and headache, several common biological factors including genetic predisposition, mast cell activation, platelet activating factor, impaired arachidonic acid metabolism have been suggested to play a role.^16^,^23^-^25^

The relationship between asthma, migraine-type headaches and daily headaches have been reported in several studies.^14^,^16^-^18^,^26^-^28^ However, in the majority of these studies, diagnosis of asthma is not definitive, and is solely based on medical history of the patients, and asthma symptoms. On the other hand, the present study was carried out on patients who were followed with asthma diagnosis. Consistent with the literature data, our results indicate that the frequency of migraine-type headache is higher in asthma patients.

Platelet activating factor and substance P substance are considered to function in asthma pathogenesis, and induction of migraine-type headache.^16^ In another study, a correlation between migraine-type headache and asthma is found in only female patients.^29^ In our study, the frequency of migraine-type headache was higher in female asthma patients.

Although previous studies have also demonstrated the relationship between migraine-type headache and hay fever, rhinitis, and dermatitis,^14^,^28^ there is a limited number of study suggesting a correlation between headache and respiratory or allergic symptoms.^29^-^31^ In our study, we identified a correlation between migraine-type headache and presence of allergies. In addition, low respiratory function test results were correlated with migraine-type headache. Although the underlying causes still remain unknown, based on our study findings, we can conclude that asthma and migraine-type headache share common pathophysiological pathways.

Furthermore, drugs used for the treatment of migraine-type headache (e.g. β-blockers, salicylates, and non-steroid anti-inflammatory drugs) are known to induce asthma.^25^,^32^ In the present study, none of the patients used any medication inducing asthma. In addition, the frequency of migraine-type headache was lower in patients who used inhaled steroids. A possible explanation of this finding is that steroids are partly effective in suppressing migraine-type headache.

Previous studies on asthma patients have reported that the incidence of depression and anxiety is higher in this patient group, compared to the overall population.^9^ As a result, our results are consistent with the literature. Moreover, our findings indicate that tension-type headache, a type of primary headache, is more common in patients compared to healthy controls.

In this study, the definitive diagnosis of asthma was achieved after evaluating anamnesis, spirometric examination, and physical examination. In addition, well-established diagnostic criteria were used to diagnose headaches. Unlike previous studies, diagnosis was not based on questionnaires or medical records, and all diagnoses were done using the face-to-face interview method with the patient.

### Limitations to this study

Diagnosis of allergies was based on medical records and medical history of the patient. As we were unable to retrieve allergy test results, we were unable to include these data in the analysis.

## CONCLUSION

The frequencies of migraine-type and tension-type headaches are higher in asthma patients, compared to the overall population. Patients with migraine-type headache are more prone to allergies with worse respiratory function test results. In patients with asthma with a history of allergy, the presence of headache should be questioned, and such patients should be consulted to neurology specialists to improve their quality of life.
